# Single-Port Robotic Ureteroenteric Stricture Repair: A Retrospective Cohort Review

**DOI:** 10.7759/cureus.71262

**Published:** 2024-10-11

**Authors:** Hannah Baker, Audra Garrigan, Lucas R Wiegand

**Affiliations:** 1 Urology, Florida International University, Herbert Wertheim College of Medicine, Miami, USA; 2 Urology, University of South Florida, Tampa, USA; 3 Urology, Orlando Regional Medical Center, Orlando, USA

**Keywords:** robotic surgery, single-port robotics, ureteral stricture, ureteroenteric stricture, urologic reconstruction

## Abstract

Introduction

The objective of this study is to present a series of 16 cases utilizing single-port robot-assisted repair for ureteroenteric anastomosis stricture (UES). To our knowledge, this is the first case series recorded detailing successful single-port UES revision.

Methods

A retrospective review of all patients under a single surgeon undergoing single-port robotic revision of ureteroenteric stricture following radical cystectomy with urinary diversion at our institutions from September 2020 through July 2024 was performed. Patient demographics and perioperative outcomes were assessed.

Results

The study consisted of 3 bilateral ureteroenteric strictures and 13 unilateral strictures, more commonly on the left. Stricture length averaged 2.2 cm on the left and 2.1 cm on the right. Surgeries were performed by a single surgeon using the da Vinci SP surgical system (Intuitive Surgical, Sunnyvale, California, US). The type of stricture repair included excision and primary anastomosis (EPA) with Bricker or Wallace reconstruction or Heineke-Mikulicz (HM) repair, although one case involved the necessity of revision ileocalycostomy. Two of the 16 cases were converted from robotic to open due to extensive adhesions. The average procedure length was 265 minutes (148-440). Length of stay (LOS) ranged from 0-46 days, averaging 4.9 days. There were five postoperative complications encountered including two seromas, two incisional hernias, and an episode of urosepsis. Pre- and postoperative changes in creatinine level ranged from -0.26 to 0.3 mg/dL. No renal units were lost. No patients were readmitted following initial surgical discharge. All patients returned for follow-up; none required repeat intervention.

Conclusion

SP robotic repair of ureteroenteric anastomoses is safe and feasible. The risk of complications is low with a high chance of success and possibly a lower length of stay; more research is warranted.

## Introduction

Ureteroenteric anastomosis strictures (UES) are a complication of cystectomy and urinary diversion with a reported incidence of up to 25% [1]. Although most cases are asymptomatic, chronic flank pain and urinary tract infections can be common presentations [2]. Strictures may lead to a high-pressure obstruction with a decline in renal function, loss of the renal unit, and risk of life-threatening infection in the obstructed system. Therefore, urgent intervention is recommended. Management options include urinary diversion with stents/nephrostomy tubes, endoscopic treatment, open revision, and robotic-assisted surgery. Many patients are initially treated endoscopically with dilation and stenting, but the overall success rate is dismal, ranging from 26% to 50% [3]. For patients who require multiple endoscopic interventions, are refractory to endoscopic therapy, or desire definitive repair, open or robotic reconstructive surgery is advised. With the anticipated benefits of a minimally invasive approach, including reduced morbidity and shorter hospital stay, robotic repair of UES is a favorable comparison. Recent advances in robotic surgery, including the introduction of the single-port (SP) robot, provide additional benefits, particularly in the case of prior abdominal surgery. Only one instance of single-port robotic revision of ureteroenteric stricture has been detailed in the literature [4]. The objective of this article is to describe a series of 16 cases of single-port robotic revisions of ureteroenteric anastomosis strictures following radical cystectomy and urinary diversion.

## Materials and methods

A retrospective review of patients from a single surgeon undergoing single-port robotic revision of ureteroenteric stricture following radical cystectomy with urinary diversion from September 2020 through July 2024 was performed. At our institutions, prior to the acquisition of the single-port robot, UES repair was done in an open manner. After the single-port robot became available, all patients with UES were offered and chose a robotic approach. Patient characteristics, including age, previous urinary diversion history, creatinine levels, and stricture length, were documented. Perioperative outcomes, such as procedure duration, length of stay, presence of intraoperative or postoperative complications, and follow-up status, were assessed. Sixteen patients were identified, with a mean age of 68 years old. Two patients had a neobladder and 14 patients had an ileal conduit. Ureteroenteric strictures were bilateral in three cases with unilateral strictures being more common on the left with seven cases and right stricture presentations in six cases. Stricture length averaged 2.2 cm on the left and 2.1 cm on the right (Table 1).

**Table 1:** Patient characteristics Creatinine reference range: 0.6-1.1 mg/dL

Case #	Age	Type of Diversion	Stricture Length	Preoperative Cr (mg/dL)
1	61.88	Neobladder	2 cm (Right)	1.9
2	64.49	Conduit	2 cm (Right)	1.03
3	71.08	Conduit	3 cm (Left)	1.45
4	74.21	Neobladder	2 cm (Left), 5 cm (Right)	1.1
5	68.53	Conduit	4 cm (Left), 2 cm (Right)	3.02
6	66.78	Conduit	1 cm (Right)	1.13
7	62.19	Conduit	4 cm (Left), 1 cm (Right)	1.85
8	82.12	Conduit	1 cm (Left)	1.9
9	70.07	Conduit	2 cm (Left)	1.3
10	64.77	Conduit	1 cm (Right)	0.92
11	62.27	Conduit	1 cm (Left)	2
12	74.25	Conduit	1 cm (Left)	2.6
13	85.78	Conduit	1 cm (Left)	2.1
14	81.42	Conduit	3 cm (Left)	2.2
15	60.99	Conduit	1 cm (Right)	0.9
16	49.08	Conduit	4 cm (Right)	2

Technique

All patients had nephrostomy tubes placed into the affected renal units at least four weeks prior to repair with concurrent removal of ureteral stents if present. Cytology, cross-sectional imaging, and urography did not indicate a recurrence of malignancy in applicable cases. The length and location of stenoses were confirmed with a loopogram and antegrade nephrostogram.

After confirming the length and location of the stricture with imaging, the SP robotic approach was confirmed to be appropriate. The procedure was begun by making a 3 cm incision in the midline, left upper quadrant, or right upper quadrant location, depending on the surgeon's assessment of adequate space for the robot and the risk of encountering adhesions. Pneumoperitoneum was established and the patient was placed in steep Trendelenburg. Adhesiolysis was performed and the stricture was identified. Antegrade ureteroscopy was used as needed, as well as looposcopy. The stricture was excised unless it appeared short and unilateral, and then the Heineke-Mikulwicz technique was utilized. All cases utilized Firefly^®^ indocyanine green angiography to ensure adequate blood supply. After the stricture was excised, the conduit was stretched to the healthy ureter for direct anastomosis. The contralateral ureter was also reimplanted if there was excessive kinking or tension, or if dissection of the diseased ureter affected the normal ureter. Double J stents were left for seven days and removed at the bedside with a looposcopy. Nephrostomy tubes were clamped at the termination of the procedure and removed a few days after stent removal.

## Results

**Figure 1:**
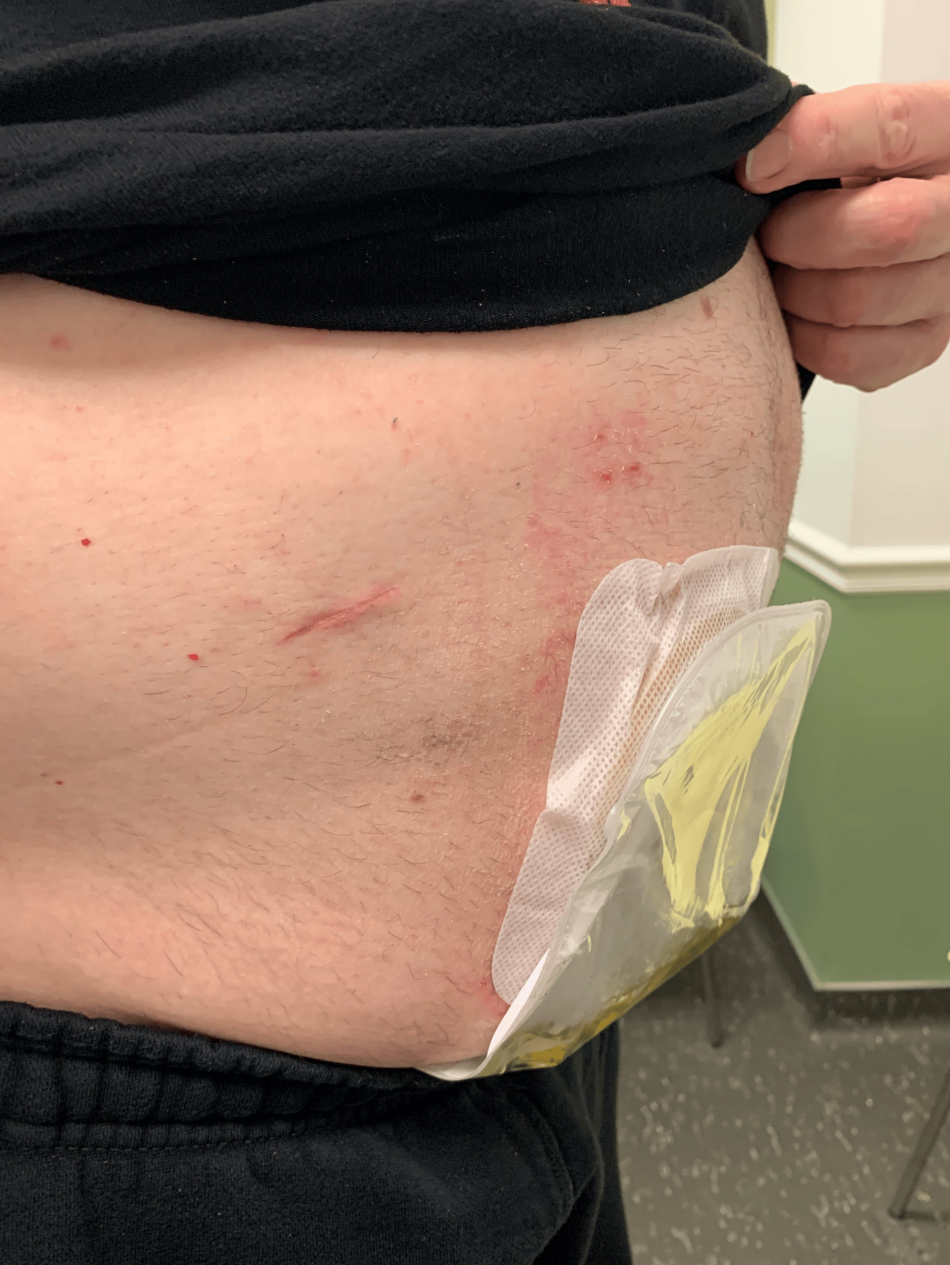
Right upper quadrant incision and ileal conduit below

All procedures were successfully completed. A midline supraumbilical port insertion site was utilized in nine of the cases, with a right upper quadrant site in six (Figure 1) and a left upper quadrant site in one. The type of stricture repair included excision and primary anastomosis (EPA) with Wallace or Bricker anastomoses or Heineke-Mikulicz (HM) tissue rearrangement. One case involved the necessity of ileocalycostomy. The first two cases were electively converted to open due to adhesions. After this, the choice of incision was preferentially the right upper quadrant. The average procedure length was 265 minutes. Length of stay ranged from 0 to 46 days, averaging 4.9 days. There were five postoperative complications encountered, including two seromas, two incisional hernias, and an episode of febrile UTI. One seroma evolved into superficial wound separation, which was treated with wound packing and healed by secondary intention. The other seroma and incisional hernias did not require operative intervention and were treated conservatively. No patients were readmitted after discharge. Pre- and postoperative changes in creatinine level ranged from -0.26 to 0.3. The mean days of follow-up was 622 (Table 2).

**Table 2:** Intra- and postoperative findings EPA - excision/primary anastomosis; HM - Heineke-Mikulicz Creatinine reference range: 0.6-1.1 mg/dL

Case #	Type of Repair	Procedure Duration (Minutes)	Length of Stay (Days)	Postoperative complications	Postoperative Creatinine (mg/dL)	Change in Creatinine (mg/dL)	Days of Follow-Up
1	EPA - BRICKER	314	4	None	1.9	0	334
2	EPA - WALLACE	257	1	None	1	-0.03	257
3	EPA - BRICKER	238	1	None	1.19	-0.26	229
4	EPA - WALLACE	292	2	None	1.36	0.26	61
5	EPA - BRICKER	353	1	None	2.79	-0.23	117
6	EPA - BRICKER	280	1	None	1.13	0	106
7	EPA - BRICKER	289	1	None	2.07	0.22	96
8	EPA - WALLACE	194	1	None	1.8	-0.10	507
9	EPA - WALLACE	165	3	None	0.8	-0.50	1235
10	HM	148	2	Urosepsis, seroma	0.74	-0.18	1468
11	EPA - WALLACE	242	2	Seroma, incisional hernia	1.6	-0.40	1417
12	EPA - WALLACE	282	1	None	2.5	-0.10	681
13	EPA - WALLACE	303	0	None	2.3	0.20	591
14	EPA - BRICKER	264	5	Converted to open - mild dehiscence (packed)	2.5	0.30	1074
15	EPA - WALLACE	192	8	Converted to open - appendectomy due to adhesion	0.85	-0.05	1165
16	EPA - ileocalycostomy	440	46	None	2.11	0.11	551

All have been followed in an outpatient setting for recurrence of symptoms, worsening creatinine levels, or evidence of obstruction on imaging. For follow-up imaging, renal ultrasound was preferred, but a nuclear medicine renal scan was used in any case of persistent hydronephrosis to rule out obstruction and confirm the preservation of renal function. No patient had a recurrence, which was defined as a return of obstruction or loss of function based on imaging or the need for any further procedure for obstruction.

## Discussion

This study demonstrates the feasibility of single-port robotic revision of ureteroenteric stricture. Ureteroenteric stricture is a known complication of radical cystectomy and urinary diversion with significant consequences if left untreated. Risk factors have been suggested, including obesity, prior abdominal surgery, and comorbidities leading to higher American Society of Anesthesiologists (ASA) scores [5], but there is insufficient evidence for a consensus. When looking at approaches to conduit construction, one study showed a higher incidence of UES associated with robotic intracorporeal diversion compared to robotic extracorporeal or open approach [6]. However, this remains controversial. Lastly, the Bricker technique was responsible for the higher number of UES compared to Wallace in one study, but this was not supported in a separate meta-analysis [1].

Currently, there exist no standard guidelines for the prevention of UES post-cystectomy. The use of indocyanine green immediately before ureteroenteric anastomosis to investigate the adequacy of the blood supply has been shown to reduce the development of UES [7,8]. Further studies should be done to investigate this strategy.

Reconstructive surgery is the gold standard for UES not amenable to endoscopic repair or for those surgically unfit for more invasive management. When comparing the open versus the robotic approach, both have a high success rate, yet robotic repair has been shown to have fewer complications [9]. Although treatment of UES using robotic-assisted surgery is more recent, studies have demonstrated the safety and efficacy of this method, leading to a shortened length of stay and benign postoperative course [9-11]. Only one patient in our cohort had a length of stay of 46 days; otherwise, patients were discharged on average between 0 and 8 days. No patients were readmitted after discharge. A similar cohort of open repairs showed a median length of stay of 6 days as compared to 4.9 days for this cohort, which could represent selection bias [12]. An encouraging finding is that for the cohort that was not converted to open and excluding the one LOS outlier, the mean LOS was 1.5 days.

Endoscopic treatment has been a mainstay of UES treatment, but results are generally not as good as formal repair. Van Son et al. showed 69% success after open repair versus 27% for endoscopic treatment at a median follow-up of 34 months [13]. It is the authors' opinion that endoscopic treatment should be reserved for patients who are not acceptable surgical risk. Others have indicated that a cutoff of 1 cm stricture length should be used for an attempt at endosurgical treatment, but even that was significantly less successful than open repair [14].

Two of the initial patients were converted due to adhesions. This was mostly thought to be secondary to the incision being in the midline. Additionally, midline incisions in the epigastric area had several postoperative hernias. For these reasons, we converted to upper quadrant incisions and were very happy with the exposure and ease of lysis of adhesions while maintaining excellent success in repair. This is now the preferred approach at our institution.

Like other open surgeries that have transitioned to robotics, single-port robotic UES repair seems to offer improved cosmesis (Figure 1) and reduced pain, making it an attractive option for appropriate patients. This has been shown in the pyeloplasty population when comparing multi-port to single-port repairs [15]. Whether the pain has actually improved or patient satisfaction improved regarding cosmesis or other factors was not determined in this article, but it may be a future direction. Our 16 cases demonstrated that single-port robotics is a viable option for the treatment of UES, with a low rate of postoperative complications and a high success rate in short-term follow-up.

The limitations of our study include a small sample size without a comparison cohort. As the popularity of the single-port system rises, future studies should be completed to identify differences between multiport and single-port treatment of UES.

## Conclusions

Single-port robotic-assisted surgery is a safe and effective method for the treatment of UES. All patients were successfully treated with minimal postoperative complications and no readmission. To date, no patient has had a recurrence of obstruction. This approach suggests a promising future for minimally invasive urologic reconstructive surgery.
